# netgsa: Fast computation and interactive visualization for topology-based pathway enrichment analysis

**DOI:** 10.1371/journal.pcbi.1008979

**Published:** 2021-06-11

**Authors:** Michael Hellstern, Jing Ma, Kun Yue, Ali Shojaie

**Affiliations:** 1 Department of Biostatistics, University of Washington, Seattle, Washington; 2 Public Health Sciences Division, Fred Hutchinson Cancer Research Center, Seattle, Washington; Johns Hopkins University, UNITED STATES

## Abstract

Existing software tools for topology-based pathway enrichment analysis are either computationally inefficient, have undesirable statistical power, or require expert knowledge to leverage the methods’ capabilities. To address these limitations, we have overhauled NetGSA, an existing topology-based method, to provide a computationally-efficient user-friendly tool that offers interactive visualization. Pathway enrichment analysis for thousands of genes can be performed in minutes on a personal computer without sacrificing statistical power. The new software also removes the need for expert knowledge by directly curating gene-gene interaction information from multiple external databases. Lastly, by utilizing the capabilities of Cytoscape, the new software also offers interactive and intuitive network visualization.

## Author summary

With the increase in publicly available pathway topology information, topology-based pathway enrichment methods have become effective tools to analyze omics data. While many different methods are available, none are uniformly best. This paper focused on overhauling an existing topology-based method, NetGSA. The three key improvements included dramatically reduced computation time so pathway enrichment can be performed within minutes on a personal computer, integration of publicly available pathway topology databases so users can easily leverage the entire capabilities of the NetGSA method, and facilitating interactive visualization of results through an interface with Cytoscape, a popular network visualization tool. The improved NetGSA was compared to the previous version as well as other similar pathway topology-based methods and achieves competitive statistical power. With these improvements and NetGSA’s flexibility to address a diverse set of problems and data types, we believe that the new NetGSA can be a useful tool for practitioners. The updated NetGSA is available on CRAN at https://cran.r-project.org/web/packages/netgsa/index.html and the development version is available on GitHub at https://github.com/mikehellstern/netgsa.

This is a *PLOS Computational Biology* Software paper.

## Introduction

Pathway enrichment analysis methods have become standard tools for analyzing omics data [1]. While earlier generations of methods are still widely used, the third generation, topology-based methods, may offer advantages by incorporating the pathway structure [2]. Despite these advantages, limitations in existing methods and software have hindered wide adoption of topology-based methods [1]. To overcome these limitations, this paper provides important updates to an existing statistically powerful method, NetGSA [3, 4], which was previously difficult to use and computationally expensive [5].

Many topology-based methods have been proposed in the literature, but there is no consistent best choice for a given problem. For example, computationally efficient methods, such as SPIA [6] and PRS [7] require differentially expressed genes which may or may not be detected. Methods such as topologyGSA [8] and Pathway-Express [9] have specific input requirements and thus may not be applicable to, e.g., metabolomics data [5]. The current version of NetGSA, a statistically powerful method, is computationally slow. Finally, a major hurdle common to all topology-based methods is their reliance on external information [1]. This information is often spread across several databases, such as KEGG [10], Reactome [11], and PantherDB [12], making aggregation challenging for non-expert users.

**Fig 1:**
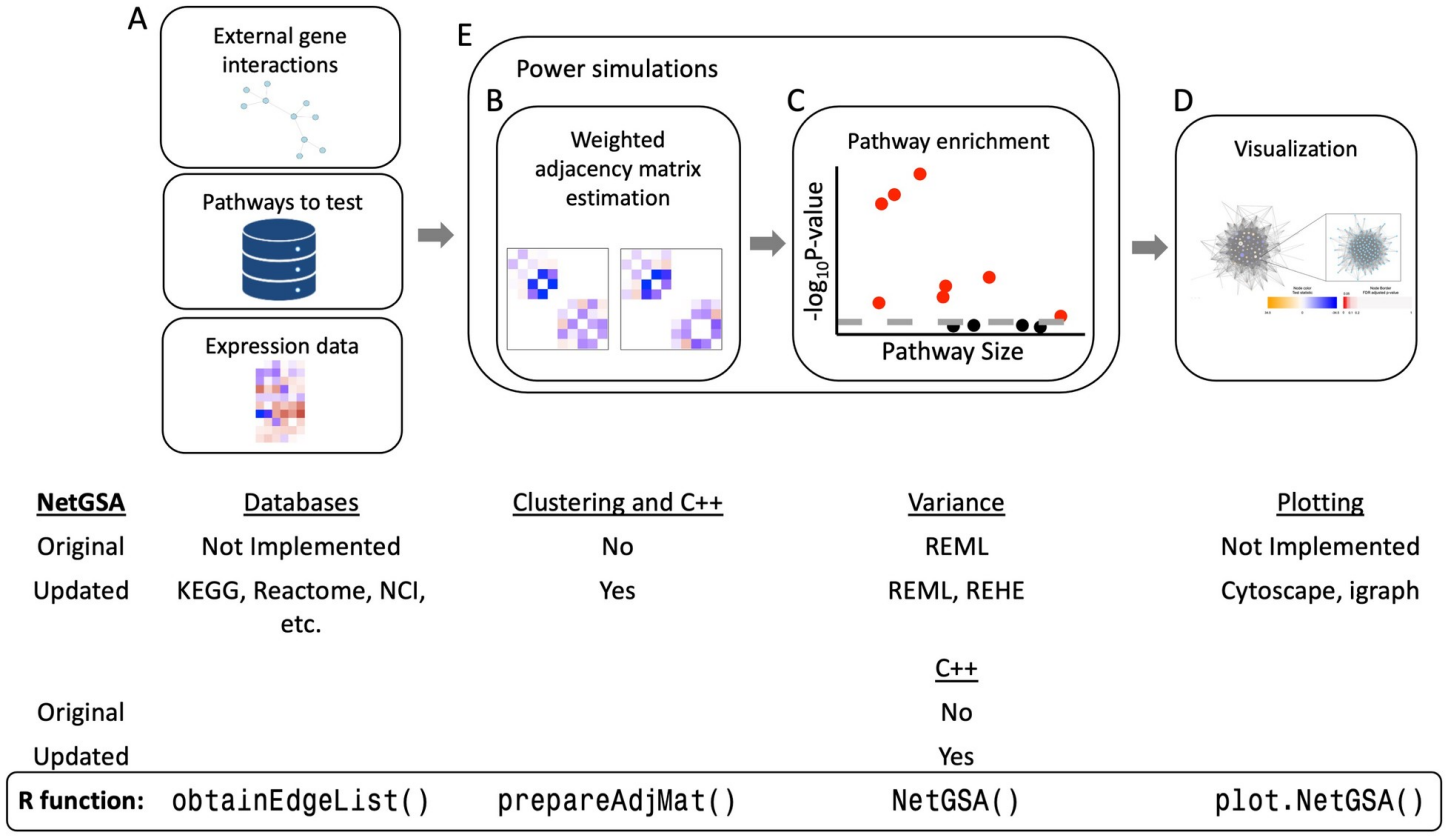
NetGSA methodology highlighting package updates. (A) NetGSA takes external gene interactions, a matrix of pathways to test, and expression data as inputs. The new netgsa incorporates existing external databases such as KEGG, Reactome, NCI, etc. (B) Weighted adjacency matrices are calculated for each condition (two conditions shown). Users now have the option to use clustering in calculating the weighted adjacency matrices. (C) Pathway enrichment is performed using weighted adjacency matrices. In addition to REML, users can estimate the variance parameters using REHE. Faster matrix calculations are also incorporated in C++ (B & C). (D) Visualization is now available with the option to use either Cytoscape or igraph. (E) Power simulations involved steps (B) and (C).

These issues present a clear need for computationally efficient, statistically powerful, and user-friendly software. To address this need, we have completely overhauled the netgsa R package, an implementation of the NetGSA methodology. In addition to desirable statistical power, NetGSA has a number of appealing features. Most notably, it provides a flexible framework for testing pathway enrichment in complex experiments [13] and diverse data types, and is robust to errors or incompleteness of existing biological network databases [3]. However, the implementation of the method in the netgsa package was overly complicated, requiring users to manually extract and supply biological network information. The package was also prohibitively slow for analyzing data with large numbers of omics measures. To address these shortcomings, we have drastically simplified the netgsa workflow to three functions and netgsa now connects seamlessly with several knowledge bases and interactive visualization tools to vastly improve the user experience. netgsa’s computation has also been significantly improved and pathway enrichment can now be performed in minutes on a personal laptop with no loss in statistical power. Fig 1 gives an overview of the NetGSA methodology and changes in the netgsa software.

## Design and implementation

The updated version of netgsa includes important user interface improvements, streamlining the workflow from analysis to visualization. The package also continues to support diverse omics data types, including gene expression, proteomics and metabolomics data sets. However, to simplify the presentation, we describe the netgsa’s features in the context of gene expression data sets.

### User interface: Input

The inputs to netgsa are gene interactions (optional), a list of pathways to test, and gene expression data. Gene interaction information is an optional input as netgsa can directly learn the network from expression data. However, this information is recommended for leveraging the full power of the NetGSA method. Previously, users had to manually supply this information. This was a time consuming task as there are numerous fragmented databases each using different identifiers. netgsa now interfaces directly with graphite [14] to search for interaction information so users can easily access all of netgsa’s functionality. Users can specify any of NDEx [15] or the eight pathway databases available in graphite—KEGG [10], BioCarta [16], Reactome [11], NCI/Nature Pathway Interaction Database [17], PathBank [18], PantherDB [12], smpdb [19], PharmGKB [20]—and netgsa will retrieve the interaction information. This functionality is available with a single R function: obtainEdgeList().

**Fig 2:**
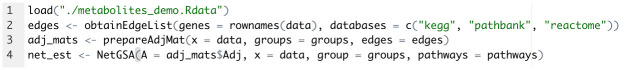
The new netgsa workflow in R.

The next step in the netgsa workflow is to estimate the weighted adjacency matrices for each condition using the interaction network collected. The new prepareAdjMat() function now detects the network type (directed/undirected), compiles the interaction information for users, and estimates the weighted adjacency matrix for each condition. With these improvements, pathway enrichment can be performed with three simple functions as shown in Fig 2. Alternatively, NetGSAq() can also be used to perform weighted adjacency matrix estimation and pathway enrichment in a single function call.

### User interface: Output

Pathway enrichment analysis typically consists of large biological networks which are difficult to visualize. Static images are either too specific, offering only a local view, or too broad, yielding incomprehensible pictures. To remedy this, netgsa now connects directly with Cytoscape [21], a Java-based interactive network visualization tool.

**Fig 3:**
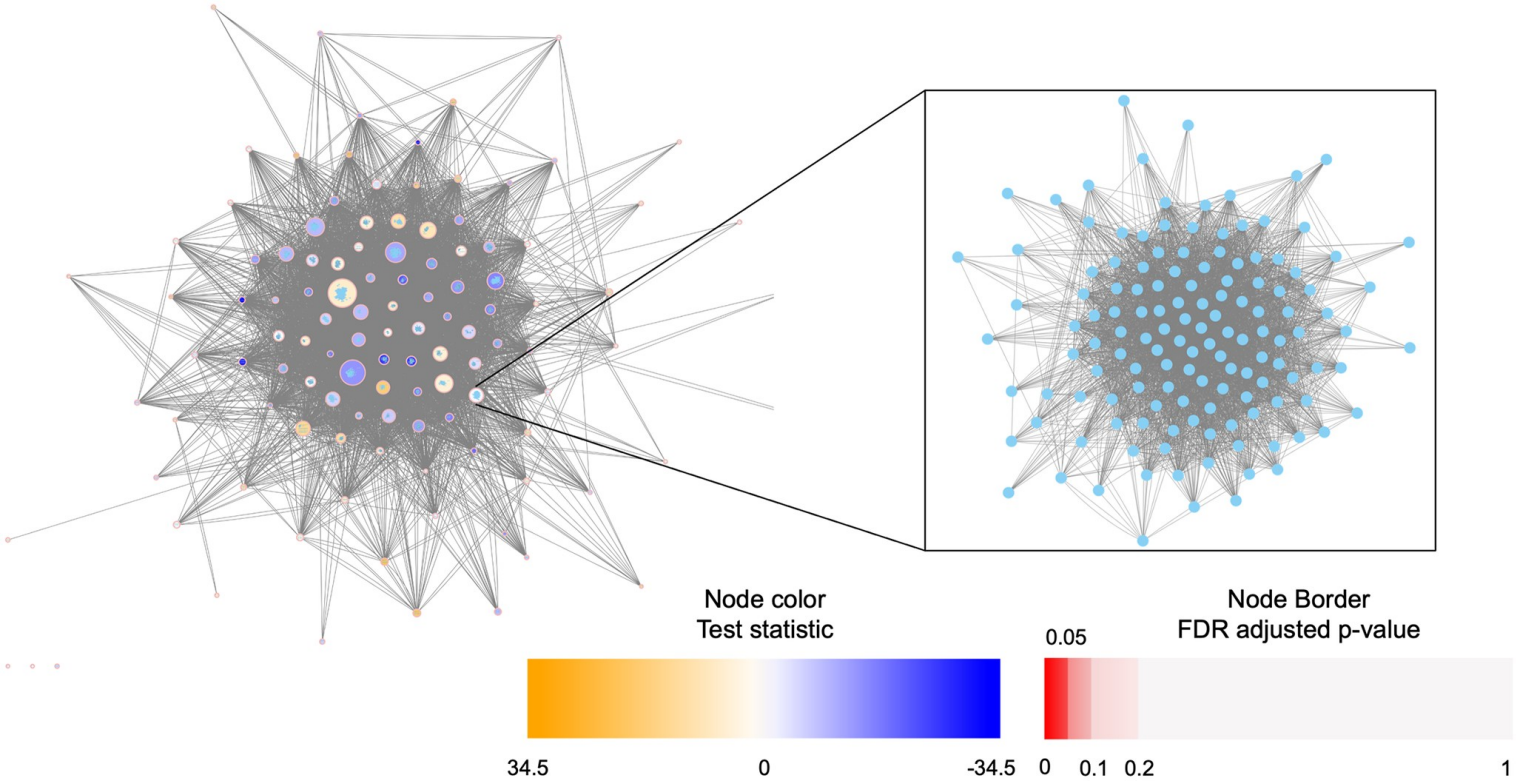
Example of a nested network Cytoscape plot for breast cancer data.

Cytoscape offers an intuitive and user-friendly interactive display. Users simply need to have Cytoscape installed and running and use the plot() function in netgsa to generate visualizations in Cytoscape. The default network plot displays pathways as nodes and between pathway interactions as edges. Two pathways are connected if at least one gene from each pathway are connected. By default, pathway nodes are colored according to both FDR adjusted *p*-values and values of the test statistic returned by NetGSA(); all data are loaded into Cytoscape, so further customization is available for users familiar with the software. Additionally, the subnetwork containing only statistically significant pathways is also plotted to reduce visual complexity. The visualizations use Cytoscape’s nested network format, so users can easily zoom-in to see the pathway members and their interactions. See Fig 3 for an example visualization produced by netgsa in Cytoscape based on breast cancer gene expression data from The Cancer Genome Atlas [22]. When Cytoscape is not open or is unavailable, the network is plotted using the igraph package in R.

### Computational improvements

The new version of netgsa couples user interface improvements with computational advances. Users can now choose the Restricted Haseman-Elston (REHE) method (set as default) [23] to estimate the variance components of the latent variable model for inference. Previously, the variance components were estimated by Restricted Maximum Likelihood (REML) based on a Gaussian likelihood. This maximization procedure required computing the gradient and Hessian of the profile log-likelihood, a computationally expensive procedure. By using a constrained method of moments estimator, the REHE method is much more efficient than REML. The increase in efficiency is particularly salient for large data sets. Additionally, users can estimate the adjacency matrices via the cluster option (set as default) in prepareAdjMat(), which is referred to as “clustering” in this paper.

**Fig 4:**
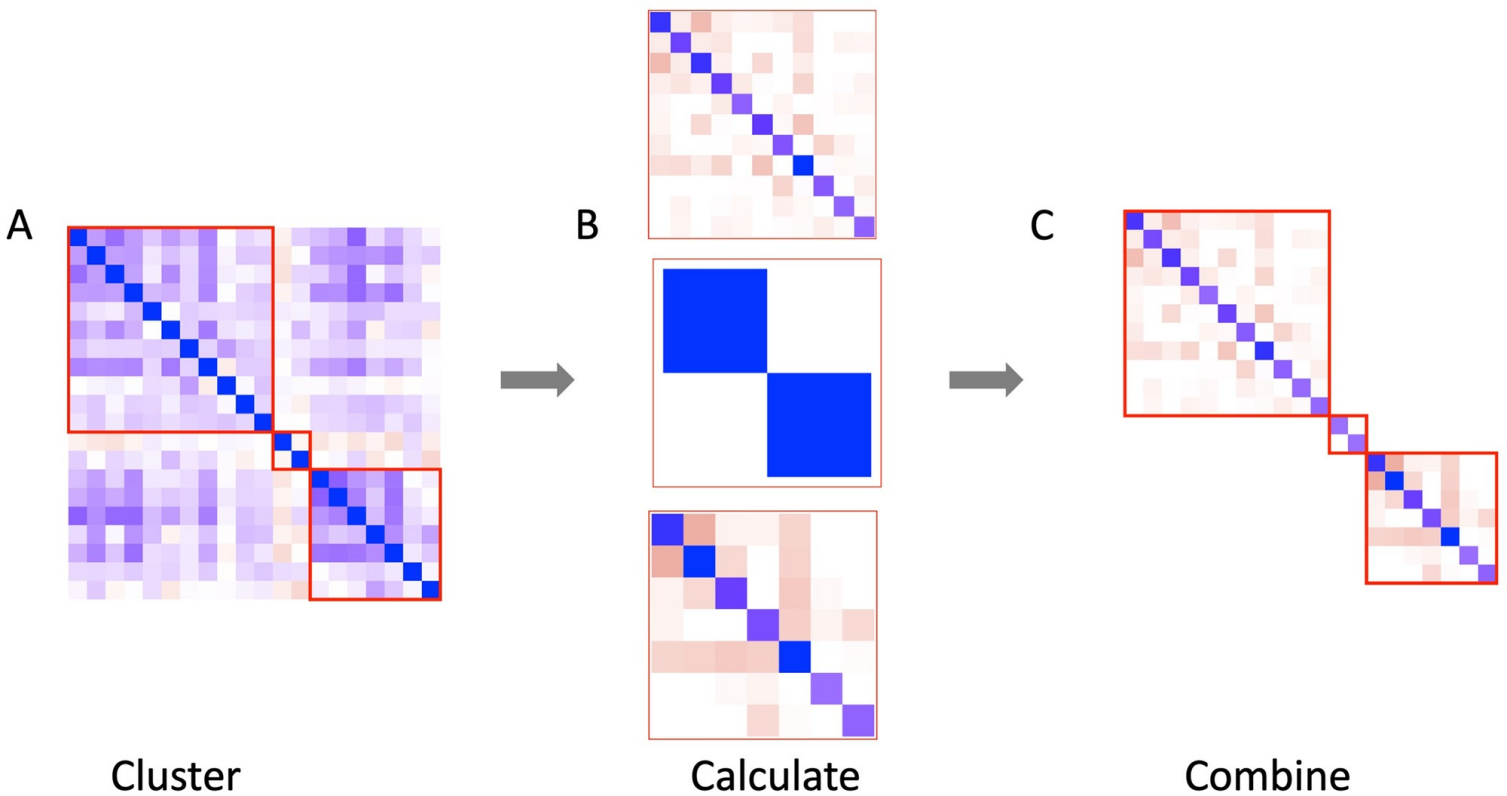
Illustration of block diagonalization and clustering in calculating weighted adjacency matrix. (A) The network is broken into clusters. (B) Weighted adjacency matrix estimation is performed on each cluster separately. (C) Cluster specific matrices are recombined into a single matrix.

To cluster genes, the 0–1 adjacency matrix from the gene interactions in e.g. obtainEdgeList() is used to identify connected components in the network. Next, six clustering algorithms from the igraph package, specifically cluster_walktrap, cluster_leading_eigen, cluster_fast_greedy, cluster_label_prop, cluster_infomap, and cluster_louvain, are run on each connected component >1,000 genes. In order to ensure clustering reduces computational complexity, algorithms producing a maximum cluster size >1,000 genes are discarded and among the remaining, the algorithm with the smallest edge loss is chosen. Weighted adjacency matrices are estimated for each cluster and reassembled into a block diagonal matrix as the final estimate of the weighted adjacency matrix for the entire network. By leveraging this block diagonal structure, clustering can reduce the computational complexity of estimating the weighted adjacency matrix by orders of magnitude; see Fig 4 for an illustration. The new version of netgsa also incorporates much faster matrix calculations in C++ through the RcppEigen package.

## Results

## Power analysis

Reducing the network to a block diagonal structure through cluster detection and efficient estimation of variance components dramatically reduces the computation time. However, these improvements would be useless if they lead to diminished statistical power or inflated type-I error.

To evaluate the statistical power of the new netgsa software, the datasets and dysregulation frameworks in [5] were used to estimate statistical power with and without clustering. There were a total of three datasets, two of which come from The Cancer Genome Atlas (TCGA). The first involves an analysis of primary breast cancers. Gene expression data was collected for 2784 genes on 520 samples, of which 403 were estrogen-receptor-positive (ER+) and 117 were estrogen-receptor-negative (ER-) [22]. The pathways tested were 114 signaling and metabolic pathways from KEGG [10]. The second comes from a prostate cancer study which measured gene expression of 2952 genes for 264 case and 160 control subjects [24]. For this data, 112 KEGG signaling and metabolic pathways were analyzed. The final dataset is much smaller and measured metabolic profiles among 41 non-diabetic and 30 diabetic mice for 100 selected metabolites [25].

To introduce artifical signal to estimate the statistical power, data for each gene or metabolite was centered and scaled to have zero mean and unit variance. A subset of genes or metabolites were chosen to be dysregulated based on three different dysregulation frameworks detailed in [5]. If a gene was dysregulated, a mean signal was added for a chosen condition (e.g. in the breast cancer data no signal was added to data from ER- subjects, but the mean signal was added to data from ER+ subjects). Three different mean signals were used: 0.2, 0.3, 0.4. Pathways with at least one dysregulated gene were used to assess power and those with no dysregulated genes were used to evaluate type-I error. An FDR cut-off of *α* = 0.05 was used to identify statistical significance. Due to computational considerations, REML power calculations were performed on each pathway separately, while REHE calculations were performed on the entire network. As an additional comparison, REHE was also run on each pathway separately and achieves similar power to REML by pathway (S1 Appendix). However, this approach may not offer significant reduction in computational time compared with REHE with clustering. Therefore, we recommend using NetGSA with REHE and clustering options.

**Fig 5:**
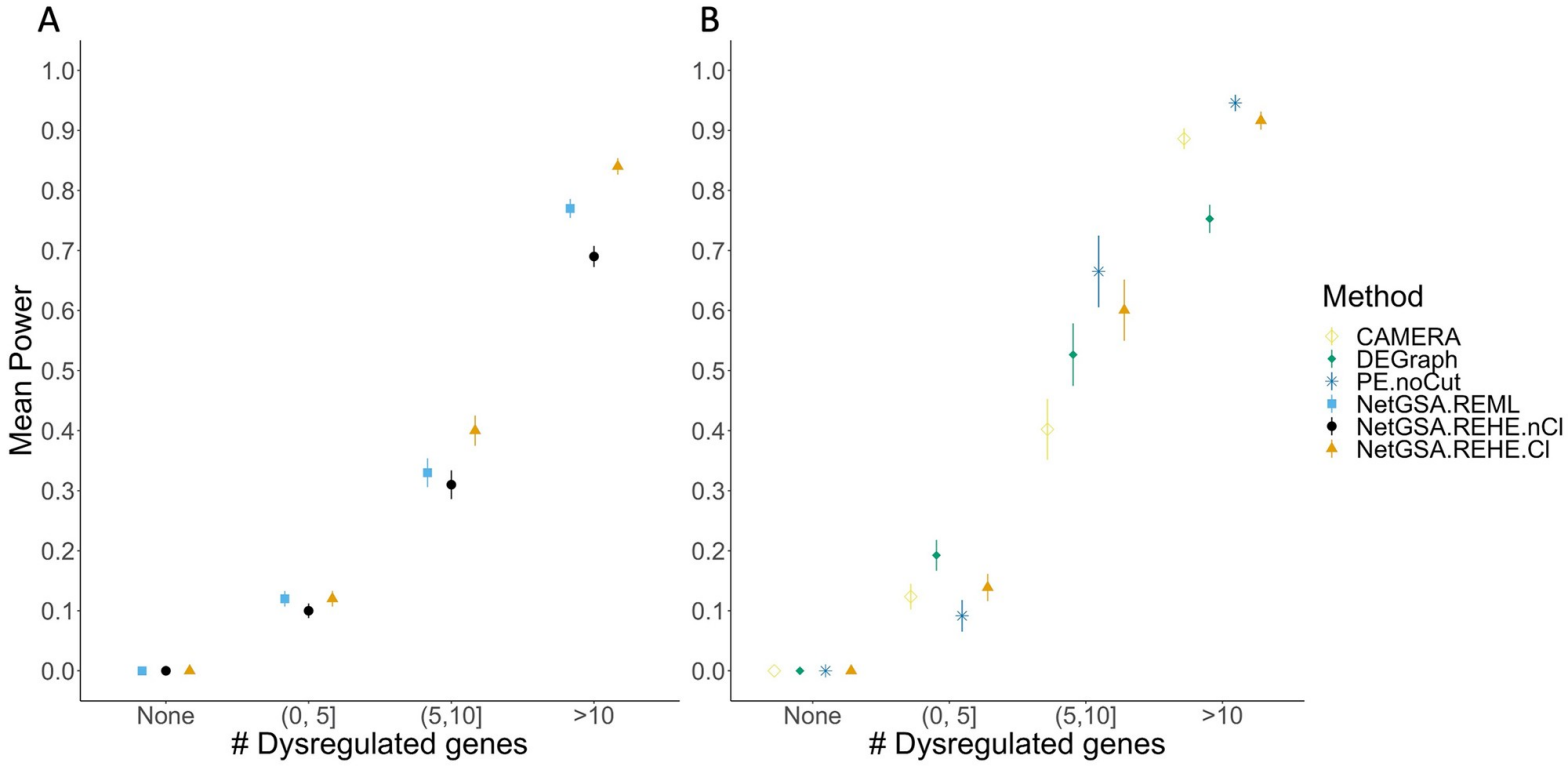
(A) Power analysis for all datasets and all mean dysregulations, grouped by number of dysregulated genes in pathways; the “None” group corresponds to null pathways. (B) Power comparison for all mean dysregulations for breast cancer data. The methods considered were CAMERA; DEGraph; PE.noCut (Pathway-Express without cut-offs); NetGSA.REML (NetGSA with REML); NetGSA.REHE.Cl (NetGSA with REHE and clustering); and NetGSA.REHE.nCl (NetGSA with REHE and no clustering).

Each pathway was grouped based on its number of dysregulated genes: None, (0,5], (5,10], >10. For example, the Galactose metabolism pathway tested in the breast cancer dataset had 4 dysregulated genes under the betweeness dysregulation framework [5]. Power estimates were averaged over all pathways and datasets for each group and are displayed in Fig 5A along with the standard errors. The “None” group shows a mean power of 0 suggesting that type-I error is well controlled in all settings. For all other numbers of dysregulated genes, mean power is higher using REHE with clustering compared to both REHE without clustering and REML. Standard errors across the methods also appear to be comparable. Similar results were obtained when analyzing power for each level of dysregulation and aggregating based on pathway size rather than number of dysregulated genes, with power increasing as mean dysregulation and pathway size increase respectively (S1 Appendix).

In addition to comparing against the old software, Fig 5B compares the recommended new netgsa software, i.e., NetGSA with REHE and clustering (NetGSA.REHE.Cl), to existing methods using a similar methodology to [5]. Specifically DEGraph [26], CAMERA [27], and Pathway-Express without cut-offs [9] were considered. These methods were chosen as they are not restricted in their input requirements. Over-representation analysis (ORA) type methods were excluded from this analysis as they require a list of differentially expressed genes, assessed using an FDR adjusted two sample t-test with unequal variances, which were almost never detected for low dysregulation values (0.2, 0.3). A similar finding was reported in [5]. The excluded ORA-type methods were Pathway-Express with cut-offs [9], PRS [7], SPIA [6], and CePA ORA [28]. Three other methods, topologyGSA [8], PathNet [29], and CePa with gene set analysis [28] were excluded for other reasons: topologyGSA requires the pathway topology to be a directed acyclic graph (DAG); PathNet has been shown to have uncontrolled type I error [5]; and CePa with gene set analysis had very low statistical power (near 0) for all dysregulation groups which is likely due to the low levels of mean dysregulation used in our simulations. Due to time considerations, the selected methods were only compared using the breast cancer dataset outlined above. Similar to [5], all methods control the type I error. DEGraph performs well for pathways with fewer dysregulated genes, but relatively worse for pathways with more dysregulated genes. Conversely, CAMERA and PE.noCut perform relatively better for pathways with higher numbers of dysregulated genes, but relatively worse for pathways with fewer dysregulated genes. The new netgsa software, NetGSA.REHE.Cl, performs favorably in terms of power and is competitive with the top method across all dysregulated gene groups.

### Computational time

The power analyses performed in the previous section were run in parallel on a cluster with 4 nodes each with two 10-core CPUs and 128GB of memory. For the netgsa comparison, netgsa was also timed using REML on the entire network to estimate the variance components (NetGSA.REML.All). This is different than the power results presented in Fig 5 which were estimated using REML for each pathway (NetGSA.REML). This difference is because calculating power for REML on the entire network was too computationally expensive, so only 10 iterations for each dataset were timed. The mean and standard deviation of the combined run time for network estimation (prepareAdjMat()) and pathway enrichment (NetGSA()) are shown in Table 1 for the breast cancer and prostate cancer datasets from [5]. Estimation with REHE is several times faster than REML and clustering provides an additional large improvement. Separate timing results for network estimation and pathway enrichment are given in S1 Appendix.

**Table 1:** Timing results (in minutes) for NetGSA with REML and REHE with and without network clustering for both the prostate and breast cancer datasets.

Method	Mean	SD
NetGSA.REML.All	167.83	30.69
NetGSA.REHE.nCl	33.35	14.12
NetGSA.REHE.Cl	4.60	1.18

The computational timing of netgsa is compared with other existing methods in Table 2. While faster than the old netgsa, the new software is not as fast as the other methods tested, reflecting a potential area for future improvement. It is worth noting that even though netgsa is still relatively slow, it is now much closer in absolute time to the other methods tested. A chief portion of netgsa’s computational time is driven by the estimation of the weighted adjacency matrix. While inherently time consuming, this step offers robustness to noise or incompleteness of externally-obtained network information. It also allows netgsa to assess changes in both mean expression levels as well as network connectivities [3, 13]. Thus, the additional computational time offers appealing features not provided by other existing approaches. Furthermore, netgsa now comes with additional features from visualization to user interface improvements. Finally, netgsa offers additional flexibility for multi-condition studies (e.g., more than 2 conditions), and can directly incorporate additional covariates.

**Table 2:** Timing results (in minutes) comparing NetGSA with REML and REHE to select methods for breast cancer data.

Method	Mean	SD
CAMERA	0.0018	0.0002
DEGraph	0.0235	0.0007
PE.noCut	0.5162	0.0237
NetGSA.REHE.Cl	4.9464	1.1530

## Availability and future directions

The updated netgsa package offers important computational and user interface improvements. It obtains external pathway information from a variety of databases automatically, solving the problem common to many pathway topology-based methods; it also offers intuitive visualizations using the capabilities of Cytoscape. Furthermore, by using network clustering and REHE for estimating the variance components, netgsa is no longer prohibitively slow. Pathway enrichment analysis with ∼ 2, 500 genes can be performed in minutes on a personal laptop with no loss in statistical power or control of type-I error. With these improvements, we believe netgsa can now be a useful tool for practitioners, especially when available network information may be noisy or incomplete, or when performing more complex, multi-condition pathway enrichment analysis. The updated NetGSA is available on CRAN at https://cran.r-project.org/web/packages/netgsa/index.html and the development version is available on GitHub at https://github.com/mikehellstern/netgsa.

## Supporting information

S1 Appendix Supporting tables and discussion. (PDF)
