# Supplementary material for: netgsa: Fast computation and interactive visualization for topology-based pathway enrichment analysis
Source: PLoS Comput Biol. 2021 Jun 11;17(6):e1008979. doi: 10.1371/journal.pcbi.1008979 (PMC8221786; doi:10.1371/journal.pcbi.1008979)
Supplement: S1 Appendix — (PDF) [file pcbi.1008979.s001.pdf]

Table A displays the numerical results of the power estimates shown in Fig 5A. These power analyses are aggregated over all datasets (breast cancer, prostate cancer, and metabolomics). Table B provides a legend and references for all methods studied. Table C displays the power estimates for `netgsa` compared to all other methods and reflects the numbers of Fig 5B presented in the main paper showing that the new `netgsa` is competitive in terms of power. Power estimates for NetGSA with REHE performed on each pathway separately (NetGSA.REHE.perPath) was also included as an additional comparison. These results show that per pathway analysis leads to a slight decrease in estimated power compared to clustering.

Table D displays the timing results for both the network estimation and pathway enrichment step in addition to the total time shown in Table 1. As mentioned in the paper, only 10 iterations were run for REML due to time considerations. Clustering results in lower computation time for both steps, but the large majority of computational gains come in the network estimation step. This is expected since clustering was designed to speed up the estimation of the weighted adjacency matrix. Computation time for pathway enrichment also decreases slightly because some calculations can be performed on each block in the clustered weighted adjacency matrix rather than the entire matrix.

Tables E, F, G show the power results for each mean dysregulation level used. The results are consistent with those presented in Table A. As expected, power increases as mean dysregulation and number of dysregulated genes within a pathway increase. Power with clustering is comparable to without across all dysregulation levels and number of dysregulated genes. Type I error remains well controlled across all mean dysregulation levels. Table H shows power results aggregated by pathway size. The results show a similar story: REHE with clustering achieves the best performance.

Table A. Power analysis for all datasets and all mean dysregulations, grouped by number of dysregulated genes in pathways; the "None" row corresponds to null pathways

# Dysregulated genes	Method	Mean	SE
None	NetGSA.REML	0.00	0.00
	NetGSA.REHE.nCl	0.00	0.00
	NetGSA.REHE.Cl	0.00	0.00
(0, 5]	NetGSA.REML	0.12	0.01
	NetGSA.REHE.nCl	0.10	0.01
	NetGSA.REHE.Cl	0.12	0.01
(5,10]	NetGSA.REML	0.33	0.02
	NetGSA.REHE.nCl	0.31	0.02
	NetGSA.REHE.Cl	0.40	0.02
>10	NetGSA.REML	0.77	0.02
	NetGSA.REHE.nCl	0.69	0.02
	NetGSA.REHE.Cl	0.84	0.01

Table B. Overview of methods

Method	Parent Method	Notes	Reference
CAMERA	CAMERA		[27]
CePa.GSA	CePa	Gene set analysis	[28]
DEGraph	DEGraph		[26]
PE.noCut	Pathway-Express	Cut-off free analysis	[9]
NetGSA.REML	NetGSA	REML for each pathway	[3], [4]
NetGSA.REHE.nCl	NetGSA	REHE without clustering	[3], [4]
NetGSA.REHE.Cl	NetGSA	REHE with clustering	[3], [4]
NetGSA.REHE.perPath	NetGSA	REHE for each pathway	[3], [4]

Table C. Power comparison for breast cancer data, grouped by number of dysregulated genes in pathways; the "None" row corresponds to null pathways

# Dysregulated genes	Method	Mean	SE
None	CAMERA	0.00	0.00
	DEGraph	0.00	0.00
	PE.noCut	0.00	0.00
	NetGSA.REHE.Cl	0.00	0.00
	NetGSA.REHE.perPath	0.00	0.00
(0, 5]	CAMERA	0.12	0.02
	DEGraph	0.19	0.03
	PE.noCut	0.09	0.03
	NetGSA.REHE.Cl	0.14	0.02
	NetGSA.REHE.perPath	0.14	0.02
(5, 10]	CAMERA	0.40	0.05
	DEGraph	0.53	0.05
	PE.noCut	0.67	0.06
	NetGSA.REHE.Cl	0.60	0.05
	NetGSA.REHE.perPath	0.47	0.05
>10	CAMERA	0.89	0.02
	DEGraph	0.75	0.02
	PE.noCut	0.95	0.01
	NetGSA.REHE.Cl	0.92	0.02
	NetGSA.REHE.perPath	0.86	0.02

Table D. Timing results in minutes for breast cancer and prostate cancer datasets

Step	Method	Mean	SD
Network Estimation	NetGSA.REML.All	24.91	14.02
	NetGSA.REHE.nCl	28.95	12.76
	NetGSA.REHE.Cl	3.60	0.94
Pathway Enrichment	NetGSA.REML.All	142.92	25.55
	NetGSA.REHE.nCl	4.40	1.44
	NetGSA.REHE.Cl	1.00	0.26
Total	NetGSA.REML.All	167.83	30.69
	NetGSA.REHE.nCl	33.35	14.12
	NetGSA.REHE.Cl	4.60	1.18

Table E. Power analysis for all datasets and mean dysregulations of 0.2, grouped by # of dysregulated genes within pathway

# Dysregulated genes	Method	Mean	SE
None	NetGSA.REML	0.00	0.00
	NetGSA.REHE.nCl	0.00	0.00
	NetGSA.REHE.Cl	0.00	0.00
(0, 5]	NetGSA.REML	0.00	0.00
	NetGSA.REHE.nCl	0.00	0.00
	NetGSA.REHE.Cl	0.00	0.00
(5,10]	NetGSA.REML	0.01	0.01
	NetGSA.REHE.nCl	0.01	0.01
	NetGSA.REHE.Cl	0.01	0.00
>10	NetGSA.REML	0.46	0.03
	NetGSA.REHE.nCl	0.39	0.03
	NetGSA.REHE.Cl	0.58	0.03

Table F. Power analysis for all datasets and mean dysregulations of 0.3, grouped by # of dysregulated genes within pathway

# Dysregulated genes	Method	Mean	SE
None	NetGSA.REML	0.00	0.00
	NetGSA.REHE.nCl	0.00	0.00
	NetGSA.REHE.Cl	0.00	0.00
(0,5]	NetGSA.REML	0.07	0.02
	NetGSA.REHE.nCl	0.06	0.02
	NetGSA.REHE.Cl	0.09	0.02
(5,10]	NetGSA.REML	0.28	0.04
	NetGSA.REHE.nCl	0.27	0.04
	NetGSA.REHE.Cl	0.40	0.04
>10	NetGSA.REML	0.86	0.02
	NetGSA.REHE.nCl	0.76	0.03
	NetGSA.REHE.Cl	0.95	0.01

Table G. Power analysis for all datasets and mean dysregulations of 0.4, grouped by # of dysregulated genes within pathway

# Dysregulated genes	Method	Mean	SE
None	NetGSA.REML	0.00	0.00
	NetGSA.REHE.nCl	0.00	0.00
	NetGSA.REHE.Cl	0.00	0.00
(0,5]	NetGSA.REML	0.28	0.03
	NetGSA.REHE.nCl	0.23	0.03
	NetGSA.REHE.Cl	0.28	0.03
(5,10]	NetGSA.REML	0.68	0.04
	NetGSA.REHE.nCl	0.65	0.04
	NetGSA.REHE.Cl	0.80	0.03
>10	NetGSA.REML	0.97	0.01
	NetGSA.REHE.nCl	0.92	0.02
	NetGSA.REHE.Cl	0.99	0.01

Table H. Power analysis for all datasets and all mean dysregulations, grouped by pathway size

Pathway Size	Method	Mean	SE
(0,20]	NetGSA.REML	0.13	0.01
	NetGSA.REHE.nCl	0.12	0.01
	NetGSA.REHE.Cl	0.12	0.01
(20,40]	NetGSA.REML	0.08	0.01
	NetGSA.REHE.nCl	0.08	0.01
	NetGSA.REHE.Cl	0.09	0.01
(40,60]	NetGSA.REML	0.23	0.02
	NetGSA.REHE.nCl	0.21	0.02
	NetGSA.REHE.Cl	0.26	0.03
(60,80]	NetGSA.REML	0.41	0.03
	NetGSA.REHE.nCl	0.40	0.03
	NetGSA.REHE.Cl	0.52	0.03
(80,100]	NetGSA.REML	0.50	0.03
	NetGSA.REHE.nCl	0.45	0.03
	NetGSA.REHE.Cl	0.65	0.03
>100	NetGSA.REML	0.69	0.02
	NetGSA.REHE.nCl	0.60	0.02
	NetGSA.REHE.Cl	0.74	0.02
